# Hypothyroidism: Cardiovascular Endpoints of Thyroid Hormone Replacement

**DOI:** 10.3389/fendo.2019.00888

**Published:** 2020-01-09

**Authors:** Angeliki Stamatouli, Pablo Bedoya, Sahzene Yavuz

**Affiliations:** Division of Endocrinology, Diabetes and Metabolism, Virginia Commonwealth University, Richmond, VA, United States

**Keywords:** hypothyroidism, L-T4, L-T3, heart failure, blood pressure, hypertension, carotid intima-media thickness, hyperlipidemia

## Abstract

Thyroid dysfunction, either thyrotoxicosis or hypothyroidism, represents an important cardiovascular risk factor. Heart disease is the leading cause of death for men and women in the United States. Cardiovascular disease is multifactorial and many efforts have been made to assess precipitants for optimal guideline-based, primary, and secondary prevention. Thyroid hormone receptors are present in the myocardium and endothelium, and small alterations in its levels could have significant effects in cardiac function. Specifically, overt hypothyroidism is associated with an increased risk for atherosclerotic cardiovascular disease due to metabolic and hemodynamic effects. Several concomitant factors like impaired lipid profile, low-grade chronic inflammatory state, increased oxidative stress and increased insulin resistance enforce this relationship. The last decade has seen a renewed interest on the impact of subclinical hypothyroidism on the cardiovascular system and whether or not it should be treated. The aim of this review is to provide current evidence of the effect of thyroid hormone replacement, either with levothyroxine mono-therapy or in combination with liothyronine, on specific cardiovascular parameters.

## Introduction

Hypothyroidism presents in a wide biochemical and clinical spectrum from Subclinical (SH) to Overt Hypothyroidism (OH) and myxedema coma. Hypothyroidism is the most common endocrine disorder after diabetes and although standard treatment with Levothyroxine (L-T4) replacement is established as monotherapy, multiple symptoms remain unresolved despite maintaining a normal TSH. This led over the last two decades to several trials with combination treatment [Levothyroxine (L-T4)/Liothyronine (L-T3)] (1).

In a patient with intact thyroid gland, since symptoms of inadequate or lack of thyroid hormones are mostly non-specific, biochemical testing is the main diagnostic tool after excluding physiologic (like non-thyroidal syndrome) and/or testing interferences (like biotin effect). Typical signs and symptoms of hypothyroidism are correlated with the severity and duration of the disease, but are also associated with patient's age, sex, other comorbidities, and the etiology of hypothyroidism. Classic clinical findings in overt to severe hypothyroidism include: reduced deep tendon reflexes, skin changes, weight gain, reduced basic metabolic rate, cognitive dysfunction, and even hypothermia in advanced cases. Assessment of the signs and symptoms could be challenging especially if alterations in the thyroid hormone levels are subtle.

Thyroid dysfunction affects the cardiovascular (CV) system in all aspects from subclinical hypo/hyperthyroidism to overt hypo/hyperthyroidism. Several studies documented that OH and SH are associated with increased risk of coronary heart disease, atherosclerosis, and mortality (2) proportionally to the severity of thyroid failure, particularly among the patients with a TSH level ≥ 10 mIU/L.

**Figure 1:**
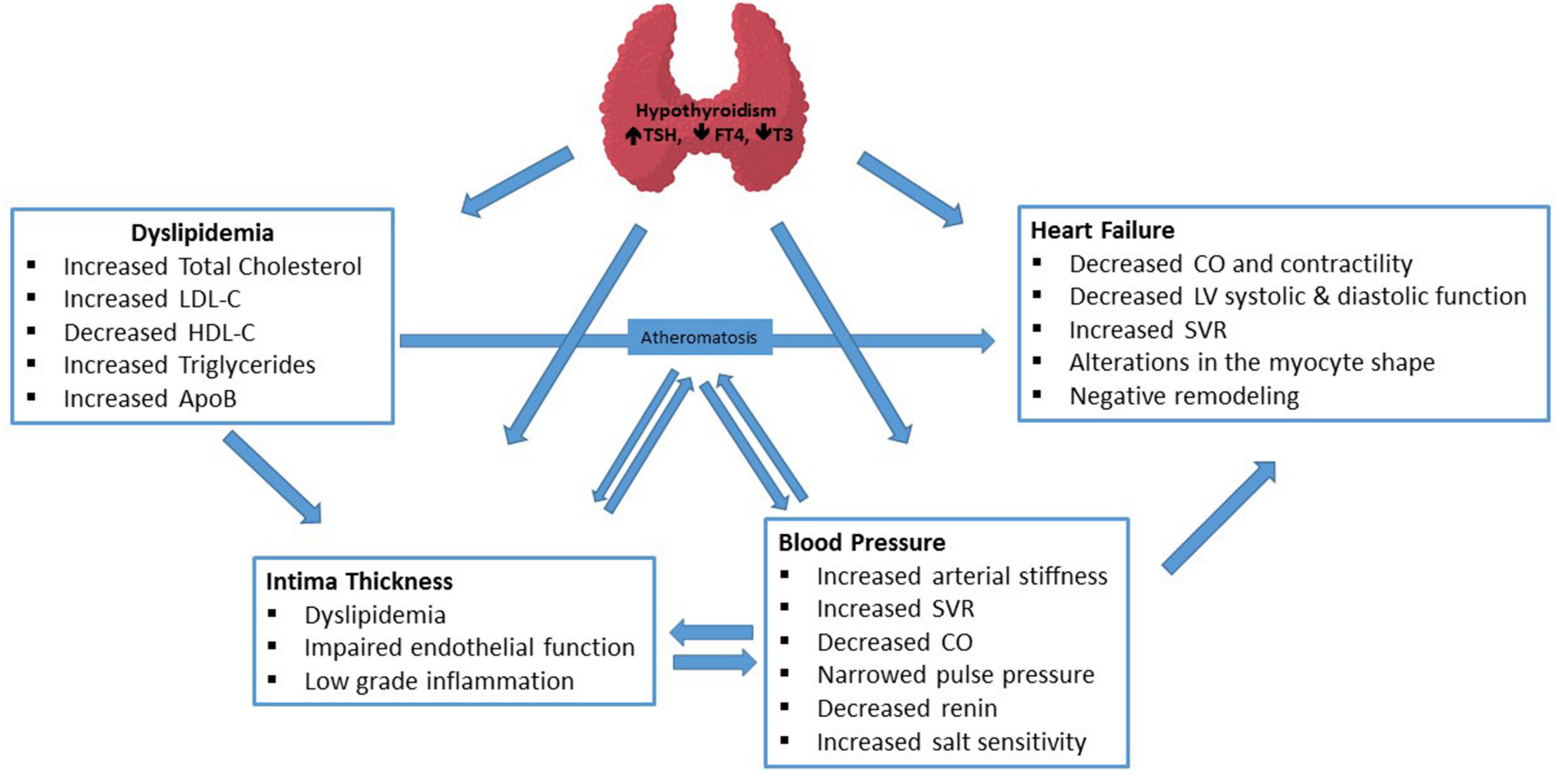
Major mechanisms of the effects of Hypothyroidism on Cardiovascular parameters.

Thyroid hormone has both genomic and non-genomic effects on CV system. While genomic actions are mediated by nuclear thyroid hormone receptors, non-genomic actions can be nuclear receptor-independent and occur at the plasma membrane level of cardiac myocytes and vasculature (3). In addition, several concomitant factors like impaired lipid profile, low-grade chronic inflammatory state, increased oxidative stress, increased insulin resistance also contribute to significant association of hypothyroidism with CV risk factors. Pathophysiologic mechanisms, briefly summarized in Figure 1, are very complex for each clinical endpoint and have been reviewed in detail elsewhere (4, 5).

The effect of thyroid hormone replacement in energy expenditure, body weight, and quality of life parameters will not be addressed in this article. In this brief review, we focused on the effect of thyroid hormone replacement on cardiovascular parameters: lipid panel, carotid intima-media thickness, blood pressure, and heart failure.

## Methods

Literature search was performed in English through PUBMED, querying for key words: hypothyroidism, thyroid hormone treatment, L-T4, L-T3, heart failure, blood pressure, hypertension, carotid intima-media thickness, and hyperlipidemia. We focused on chosen endpoints and human studies.

## Effect of Thyroid Hormone Replacement on Lipid Panel

Hypothyroidism, both overt and subclinical, could affect lipid metabolism. This relationship has been well-described in literature since 1930 and it is implicated in the increased CV risk noted on these patients. It is reported that the prevalence of hypothyroidism in patients with hypercholesterolemia is 4.3% (6). In addition, current guidelines from the National Cholesterol Education Program, the American Association of Clinical Endocrinologists, and the American Thyroid Association recommend screening for hypothyroidism the patients with newly diagnosed hyperlipidemia prior to starting a lipid-lowering agent (6–9).

OH is associated with atherogenic lipid profile with elevated levels of total cholesterol due to elevated levels of Low Density Lipoprotein (LDL) and Intermediate Density Lipoprotein (IDL), hypertriglyceridemia, and increased Apo A1-and B1-lipoprotein levels (10–12). There are multiple mechanisms involved in the pathophysiology of hyperlipidemia in hypothyroidism, such as: (a) Decreased number of LDL receptors in the liver resulting in decreased LDL uptake and accumulation (13), (b) reduced activity of the LDL receptor (14), (c) Increased LDL oxidation (15).

Hypertriglyceridemia is related to decreased activity of the lipoprotein lipase (LPL) (16), which results in decreased clearance of triglyceride rich lipoproteins and of the hepatic lipase (HL) (17), which hydrolyzes High Density Lipoprotein (HDL) and is involved in the conversion of IDLs to LDL and of LDL to small dense LDL (sdLDL). In another study, it was shown that low T3 levels are contributing to enhanced triglyceride hepatic synthesis (18).

The HDL-C levels are elevated in patients with hypothyroidism due to increased concentrations of HDL_2_ particles, reduced activity of hepatic lipase and associated decrease in the catabolism of HDL_2_s (19). In addition, reduced activity of CETP leads to reduced transfer of cholesteryl esters from HDL to Very Low Density Lipoprotein (VLDL), contributing to the higher levels of HDL-C (20).

Treatment with L-T4 significantly reduces the above documented effects in the circulating lipids. In OH, the therapy with L-T4 requires a period of 4–6 weeks to improve or correct dyslipidemia (21). Substitution treatment reduces the levels of LDL-C, total cholesterol, HDL and triglycerides, by thyroid hormone-mediated increase in the activities of lipoprotein lipase and hepatic triglyceride lipase (22). In some studies, it was shown that in patients who achieved euthyroidism with L-T4, the levels of lipoprotein A decreased (11, 12, 23). In other studies, there was no change in the levels of lipoprotein A before and after treatment with L-T4 (24, 25). Remnant lipoprotein concentrations, such as chylomicron remnants and very-low-density lipoprotein remnants, which are highly atherogenic, were lowered in patients who became euthyroid after treatment (22). For patients who received combination treatment with L-T4/L-T3, a statistically significant decrease in LDL-C and total cholesterol compared to L-T4 monotherapy was noticed after few months of treatment (26). In contrast, other trials did not show significant differences in the lipid panel of two-treatment groups (27–31).

Indeed, clinical guidelines recommend screening for hypothyroidism patients who present with dyslipidemia. It is also suggested that in patients with hypothyroidism and dyslipidemia, the administration of thyroid supplementation therapy to a statin, ezetimibe, or PCSK9 inhibitor might contribute to an enhanced and sustained effect (28, 29).

In SH, where the levels of TSH are elevated, but the levels of T4 and T3 are normal, a small percentage of patients are at risk to develop dyslipidemia, although this association is controversial, and the studies have been inconsistent. In a retrospective cohort study that aimed to determine the prevalence of thyroid function screening in patients with newly diagnosed hyperlipidemia, involving 8,795 patients, 49.5% of the patients had the thyroid function tests checked. From them, 11.1% had SH and 3.5% had TSH level of 5–10 mIU/L, suggesting that SH could be a secondary cause of hyperlipidemia and is associated with high risk of heart disease (9). Some of the studies have demonstrated that total cholesterol and LDL-C levels are elevated in patients with SH and decreased after the initiation of L-T4 (32–34). The effect of the levels of TSH to the levels of total cholesterol and LDL-C were stronger in the ages of 40–49 and 60–69 years old, compared to younger individuals, suggesting that SH might worsen the effects of aging on lipid profile (35). It was also shown that in patients with SH with higher serum TSH, there was a significantly increased serum Proprotein convertase subtilisin/kexin type 9 (PCSK9) levels which further contributes to a higher LDL-c level than the matched euthyroid participants (36, 37).

Serum HDL-C levels were found to be low (38, 39) or normal and there was no significant effect after L-T4 treatment (32, 33, 40). Serum triglyceride levels were elevated in patients with SH compared with euthyroid individuals, as it was evaluated in a meta-analysis that included 16 observational studies (41). On the contrary, other studies did not show change in triglyceride levels in SH, before and after treatment with L-T4 (10, 23, 32, 33, 40).

In SH, there is a statistically significant elevation of apolipoprotein B levels (42), which decrease after treatment with L-T4 (43). Similarly, apolipoprotein A levels were elevated in some patients with SH and decreased after the treatment with L-T4 (23). In other studies there was no significant changes in the apolipoprotein A levels and subsequent treatment with substitution therapy (32, 44). The levels of oxidized LDL-C were higher in patients with SH than in euthyroid controls and oxidative modifications of LDL-C may play a role in the initiation of atherosclerosis (45).

Currently no clinical trials reported the effectiveness of combination treatment (L-T4/L-T3) in patients with SH and dyslipidemia. Current evidence suggests that in patients with hyperlipidemia and SH, lifestyle modification, and lipid lowering medications should be started regardless of the decision for treatment with L-T4 (46).

## Carotid Intima Media Thickness

Carotid intima-media thickness (C-IMT) is a non-invasive surrogate marker of subclinical atherosclerotic alterations and used to gauge the effect of interventions that decrease atherosclerosis. In European guidelines for prevention of CV disease, C-IMT of 0.9 mm is accepted as the threshold above which atherosclerosis progression occurs. The American Heart Association/American College of Cardiology Guidelines denote C-IMT and Coronary Artery Calcium (CAC) score as a class IIa recommendation for CV risk assessment in asymptomatic adults at intermediate risk for CV disease. An increase of 0.1 mm in the C-IMT was associated with a 10–15% increase in the risk of myocardial infarction and similarly with stroke risk (47, 48). In the case of hypothyroidism, the main etiology is postulated to be the endothelial dysfunction and arterial stiffness, in addition to other factors like dyslipidemia and inflammation (4).

Two separate meta-analyses investigated the effect of LT-4 therapy on C-IMT in patients with SH. First meta-analysis (49) included nine trials (three RCT and six self-controlled study) and the second one (50) included the same nine trials plus two more. Both systematic analyses reported that LT-4 therapy reduced C-IMT significantly after long-term therapy (>6 months). The authors interpreted this effect as multifactorial, possibly due to improvement in total cholesterol, LDL-cholesterol, triglyceride levels; systolic and diastolic BP and flow-mediated dilatation with treatment. Subgroup analysis demonstrated the decrease in C-IMT was higher in subjects with baseline TSH > 10 mIU/l comparing with TSH ≤ 10 mIU/l.

A recent study compared the C-IMT in 40 OH and 30 SH female patients with euthyroid controls. In the group with OH, there was a highly significant increase in C-IMT in comparison with the control group (0.7 ± 0.2 vs. 0.45 ± 0.07 mm, *P* < 0.001), which was similar in the SH group (0.6 ± 0.2 vs. 0.45 ± 0.07 mm, *P* < 0.001). The authors also looked at the blood flow after heat-mediated vasodilation as a marker for endothelial dysfunction: comparing with euthyroid subjects there were significant impairments in both OH and SH group, more pronounced in the OH (51). Although these studies had small sample size, varied in duration and population characteristics, the signal in improvement in C-IMT was substantial and it may reflect another target in the armamentarium of modifiable CV risk factors.

A community-based study from China including 2,276 non-diabetic, euthyroid participants found a significant inverse relationship between serum free T3 levels and C-IMT (52) after excluding traditional risk factors for atherosclerosis. This is an interesting observation as most significant association was on the lower FT3 quartile, although it was still within the normal levels. Such association was also observed in a similar study that looked the association of free T4 levels and C-IMT in euthyroid subjects (53).

In contrast, another population-based cross-sectional study from Italy, involving 5,815 participants (age range 14–102 years old), did not show an association between subclinical thyroid dysfunction and increased C-IMT (54). SH group subjects were noted to have very mild thyroid dysfunction with an average TSH of 5.09 (4.41–6.84), which might have obscured subtle effects. Similarly, in an analysis of the TRUST trial, which included European population with mild SH, no significant difference in C-IMT with L-T4 treatment was found (55).

## Blood Pressure

Hypertension (HTN) is a global health problem, affecting 26.4% of adult population (56) and is one of the modifiable risk factors in CV disease morbidity and mortality. Most of the cases involved have primary HTN, but ~10% may have secondary causes, including endocrine ones. It is well-reported in literature that the incidence of HTN in cases of toxic goiter or myxedema is high and usually responds to treatment of the underlying thyroid condition (57). Specifically, hyperthyroidism is associated with systolic hypertension (58), while OH and SH with diastolic hypertension (59).

A large, cross-sectional population study of more than 30,000 patients showed a linear increase in BP with increase in TSH values even all were within the normal reference range. Comparing upper normal range of TSH (3.0–3.5) with the lower (0.5–0.99) the odds ratio for HTN was found 1.98 for men and 1.2 for women (60). Moreover, increased risk of pre-eclampsia has been reported in a study on pregnant women with SH in comparison to euthyroid women (61).

Diurnal changes occur in BP and under normal physiologic conditions a 10–20% reduction in BP occurs at night, which is called nocturnal dipping (62). Failure to show this pattern i.e., nocturnal non-dipping has been documented to be a sign of CV or metabolic complications. The loss of this nocturnal decline, i.e., the development of a non-dipping type of BP, is frequently observed in metabolic disorders and chronic kidney disease (CKD) and contributes to the development of CV disease. A recent trial reported reversal of loss of nocturnal dipping with LT-4 treatment in SH patients (63).

A meta-analysis investigating the effects of LT-4 treatment on BP in patients with SH included 29 studies (10 RCTs and 19 prospective follow-up studies) and concluded that LT-4 replacement therapy reduced the BP in the SH group significantly and may contribute to modifiable CV risk factors for these patients (64). On the other hand a large double-blind, randomized, placebo-controlled trial (TRUST) involving 737 elderly patients (65 year old or older) with SH showed no benefit from LT-4 therapy in their BP, however the BP reduction was not the primary endpoint in this study and the patient population was limited to 65 year and older individuals and cannot be generalized in all age groups or younger patients (65).

In trials with combination therapy (L-T4/L-T3), BP changes were not reported as a primary outcome measure, but as a secondary or other outcome. Two studies demonstrated no significant difference at baseline compared to combination treatment (29, 66). However, in one of them after 4 months treatment with either LT-4 or combination therapy, a reduction in diastolic BP was noticeable. Similar results were reported in another trial (in the T4 alone group) (67). Considering that these patients were not clinically and biochemically hypothyroid at the time of randomization, capturing the difference in small groups would be challenging and studies were not powered for this outcome.

In summary hypothyroidism in all spectrums, including overt and subclinical, may contribute to HTN and detailed evaluation of thyroid function is essential as part of the appropriate work-up.

## Heart Failure

Despite major improvements in medical knowledge, application of technology and new medications, CV diseases, and particularly heart failure remains one of the major causes of morbidity and mortality in the developed world. Guidelines of American College of Cardiology/American Heart Association for the diagnosis and management for heart failure recommend investigating exacerbating conditions such as thyroid dysfunction, but without specifying the impact of different TSH levels or specific T3 levels.

Several studies showed that HF patients with OH or mild thyroid dysfunction had more hospitalizations and poor prognosis, compared to patients who had normal thyroid function (68, 69). Another important observation was that about 15–30% of patients with HF reported to have low levels of T3, called “Low T3 syndrome” and this was also associated directly with the prognosis and severity of the HF (70–72).

The main findings reported in HF associated with low T3 syndrome can be summarized as: (a) Decrease in Left ventricle diastolic and systolic function, (b) Increase in systemic vascular resistance, (c) Decrease in cardiac contractility, (d) Renal function deterioration, and finally (e) cardiac output reduction. All these changes lead to alteration in cardiac myocytes and negative cardiac remodeling (5).

Reversible cardiomyopathy is documented in OH and myxedema cases and the pathophysiology includes genomic and non-genomic action of thyroid hormone in multiple levels at the myocytes and vascular system (46). The natural history of HF is characterized by progressive hypoxia, which in turn is a major driver for the ectopic expression of type-3 deiodinase, which is responsible for the shunting of T4 into reverse T3 (73). Additionally, the state of chronic inflammation associated with HF promotes the activity of type-2 deiodinase in the hypothalamus, inhibiting the release of TRH with a consequent decrease in TSH and overall thyroid hormone production. In the aggregate, these events generate a state of low T3 syndrome, which is commonly seen as an adaptive response. On the other hand, there is experimental evidence that T3 supplementation in animal models of HF has shown beneficial effects on myocardial function, suggesting that in advanced HF this condition is indeed maladaptive (4, 74).

Earlier studies showed improved cardiac output and exercise tolerance in non-ischemic HF patients with T4 replacement therapy, however they were small trials with short duration. T3 treatment in patients with low T3 levels was investigated in several studies. One of them included both patients with ischemic and non-ischemic cardiomyopathies, who were treated with T3 infusion. Their neuroendocrine profile significantly improved (heart rate, BNP, aldosterone and nor-adrenaline levels all decreased) and the ventricular performance increased (72). The study with thyromimetic DITPA (3,5-diiodothyropropionic acid), a thyroid analog treatment, did not pass phase II due to significant side effects of increased heart rate, gastrointestinal symptoms and no clear benefit in HF specific primary end points (75).

On the other hand, a European study tested T3 replacement for 3 months in chronic HF patients with low T3 levels and the primary end point of the study was left ventricular ejection fraction measured by MUGA-SPECT (76). This randomized placebo-controlled study did not support a beneficial effect of T3 treatment in this group of patients. However, one may argue that only 13 patients completed the study and their baseline ejection fraction (EF) was average 43%, so only mildly reduced. No side effects were noted in the treatment group.

Furthermore, all CV changes that occur during OH have been detected also in the SH cases, but to a lesser degree or different extent. Subtle changes in circulating thyroid hormone levels may have a significant impact on the CV system and subclinical thyroid dysfunction has been associated with a 20–80% increase in vascular morbidity and mortality risk (77–79).

In a pooled analysis of six prospective trials of HF, thyroid dysfunction was detected in more than 10% of patients, more being subclinical hypothyroid (8.1%). Both higher and lower TSH levels were associated with increased risk of HF event after adjustment of age, sex, and other risk factors (80). When a large cohort of pre-existing heart failure patients (Pen Heart Failure Study) evaluated for thyroid hormone status and CV composite endpoints, including ventricular assist device placement, heart failure, heart transplant or death, significant associations were noted: a three-fold increase in the risk of composite end point, if TSH ≥ 7and a two-fold increase, if there was isolated low T3 (81). In a study of 163 chronic HF patients with SH who were treated with L-T4 for 6 months, their physical performance was significantly improved, when normal TSH levels were reached (82).

Most recently, ThyroHeart-CHF trial is designed as a prospective, multi-center RCT to study the efficacy and safety of thyroid hormone supplementation in patients with chronic heart failure and SH. The study findings could have a significant impact on the discovery of new therapeutic targets and methods of HF (83).

## Conclusion

Thyroid hormone interacts with and influences most metabolic pathways in virtually all organ systems throughout the entire life of the organism through genomic and non-genomic actions.

Hypothyroidism has a wide spectrum of clinical manifestations. Not only major changes, but also subtle alterations in the circulating pool of thyroid hormones may cause or contribute to CV risk.

Current data is limited by small RCTs, observational and small experimental studies. Developing well-designed, large, prospective longitudinal clinical trials to identify dose-effect relationship and specific populations that can benefit from L-T4, L-T3, or combination therapy should be in the agenda of thyroid and CV health researchers.

## Author Contributions

SY and AS have made a substantial, direct and intellectual contribution to the work, and approved it for publication. PB has helped during the early literature search.

### Conflict of Interest

The authors declare that the research was conducted in the absence of any commercial or financial relationships that could be construed as a potential conflict of interest.

## Abbreviations

SH: Subclinical Hypothyroidism
OH: Overt Hypothyroidism
L-T4: Levothyroxine
L-T3: Liothyronine
CV: Cardiovascular
C-IMT: Carotid Intima-Media Thickness
CAC: Coronary Artery Calcium
RCT: Randomized Controlled Trials
CKD: Chronic Kidney Disease
BP: Blood Pressure
HF: Heart Failure
LDL: Low Density Lipoprotein
IDL: Intermediate Density Lipoprotein
HDL: High Density Lipoprotein
VLDL: Very Low Density Lipoprotein
CETP: Cholesteryl-Ester Transfer Protein
PCSK9: Proprotein Convertase Subtilisin/Kexin Type 9.
